# A review of patients discharged from Shannon Clinic- are shorter stays in secure hospitals associated with poorer patient outcomes?

**DOI:** 10.1192/bjo.2021.850

**Published:** 2021-06-18

**Authors:** Amy Grimason, Adrian East

**Affiliations:** 1 Belfast Health and Social Care Trust; 2 Southern Health and Social Care Trust

## Abstract

**Aims:**

Shannon Clinic was established as the regional secure unit in Northern Ireland in 2005 and provides medium secure care to Northern Ireland's population of 1.8 million. Previous research has shown that inpatient admissions are shorter when compared to other secure units. Northern Ireland has less secure beds per population than the other UK nations, which can be a driver for shorter hospital stays. This review was undertaken to examine if shorter inpatient stays were associated with poorer outcomes.

**Method:**

All the discharges from Shannon Clinic to the Southern Health and Social Care Trust were reviewed over a period of 10 years (2009-2019). The outcome measures examined were mortality, readmission rate and reoffending rate. Crude rates for these were calculated. To allow for comparison, these rates were compared to the systematic review findings of Fazel et al (2016), which was an international review examining patient outcomes following discharge from secure hospitals.

DUNDRUM 1 Triage Security scores for the patient group were also reviewed, to ensure a sample representative of patients needing medium secure care.

**Result:**

41 patients had been discharged during this period. DUNDRUM 1 Triage Security scores ranged from 2.44 to 3.2.

The average length of admission was 415.5 days. This is shorter than the average reported by Fazel et al (2016).

The crude rates for all of the variables calculated (mortality, readmission to hospital and reoffending) for patients discharged from Shannon to the trust were less of those reported in the systematic review by Fazel et al (2016).

**Conclusion:**

This review suggests that patient outcomes are not negatively impacted by shorter inpatient stays in secure hospitals. A possible reason for this is the regional model of care approach, which helps ensure continuity and safe management of the transition between secure care and the community. In addition, there is close multidisciplinary working with supported living providers in the trust area to ensure patients' needs are met.

Following this initial review, there are now plans to review discharge outcomes for all patients discharged during this period. There are five trust areas in total in Northern Ireland so this will allow for comparison across the region.

The review has also been used within the unit to develop information leaflets for patients at admission and posters for display in the unit. We hope this will provide clarity to patients about secure care and a sense of optimism from the start of their admission.

